# [Corrigendum] Long non‑coding RNA NEAT1 promotes ovarian cancer cell invasion and migration by interacting with miR‑1321 and regulating tight junction protein 3 expression

**DOI:** 10.3892/mmr.2024.13215

**Published:** 2024-04-03

**Authors:** Min Luo, Lei Zhang, Hongying Yang, Kaili Luo, Chen Qing

Mol Med Rep 22: 3429–3439, 2020; DOI: 10.3892/mmr.2020.11428

Subsequently to the publication of the above article, an interested reader drew to the authors' attention that, for the cell invasion and migration assay images shown for the A2780 cell line in Figs. 1 and Fig. 3 on p. 3433 and 3435 respectively, the same data panel had apparently been selected to show the results of the si-NEAT1 experiment in Fig. 1 and the si-TJP3 experiment in Fig. 3.

After having re-examined their original data, the authors have realized that the image correctly shown for Fig. 1 was inadvertently copied across to Fig. 3. The corrected version of Fig. 3, now correctly showing the data for the si-TJP3 experiment with the A2780 cell line, is shown on the next page. Note that this error did not significantly affect the results or the conclusions reported in this paper. All the authors agree to the publication of this Corrigendum, are grateful to the Editor of *Molecular Medicine Reports* for allowing them the opportunity to correct this error, and apologize to the readership for any inconvenience caused.

## Figures and Tables

**Figure 3.**
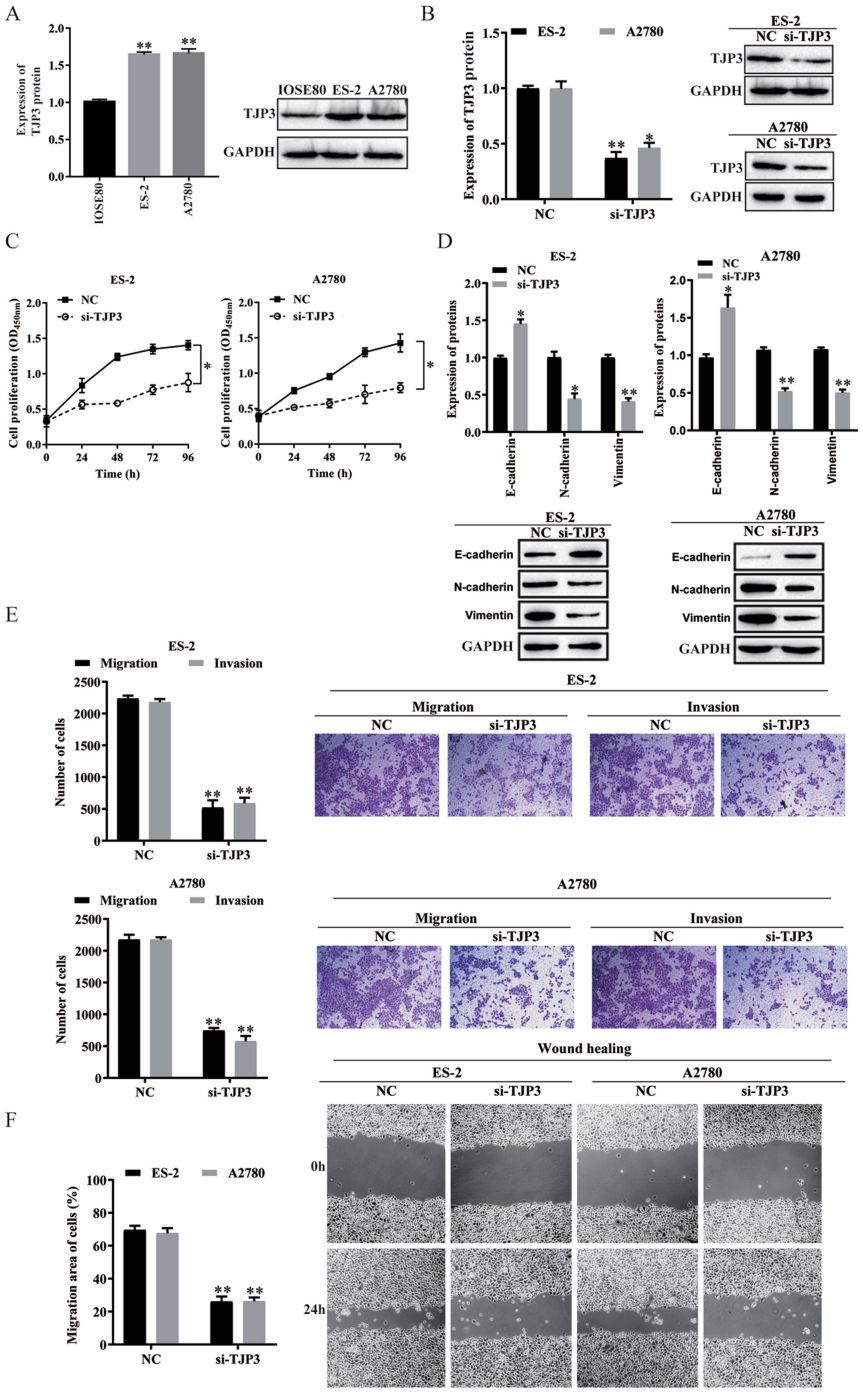
TJP3 expression and function in OC. (A) TJP3 protein expression levels in normal epithelial cells (IOSE80) and OC cells (ES-2 and A2780) were measured using western blotting. (B) Transfection efficiency of si-TJP3 in ES-2 and A2780 cells were detected using western blotting. (C) Proliferative abilities of ES-2 and A2780 cells were measured using Cell Counting Kit-8 assays. (D) E-cadherin, N-cadherin and Vimentin protein expression levels in ES-2 and A2780 cells were assessed using western blotting. (E) Migratory and invasive abilities of ES-2 and A2780 cells were measured using Transwell assays. Scale bar, 100 µm. (F) Migratory abilities of ES-2 and A2780 cells were measured using a wound-healing assay. Scale bar, 100 µm. All data are representative of ≥3 independent experiments. Data are presented as the mean ± SD. *P<0.05 vs. control group as determined by Student's t-tests or ANOVA followed by a Tukey's post hos test. TJP3, tight junction protein 3; siRNA, small interfering RNA; NC, negative control; OC, ovarian cancer; OD, optical density.

